# Caffeic Acid, an Allelochemical in *Artemisia argyi*, Inhibits Weed Growth via Suppression of Mitogen-Activated Protein Kinase Signaling Pathway and the Biosynthesis of Gibberellin and Phytoalexin

**DOI:** 10.3389/fpls.2021.802198

**Published:** 2022-01-06

**Authors:** Le Chen, Jinxin Li, Yunyun Zhu, Lujuan Guo, Rongsheng Ji, Yuhuan Miao, Lanping Guo, Hongzhi Du, Dahui Liu

**Affiliations:** ^1^Key Laboratory of Traditional Chinese Medicine Resources and Chemistry of Hubei Province, Hubei University of Chinese Medicine, Wuhan, China; ^2^National Resource Center for Chinese Materia Medica, China Academy of Chinese Medical Sciences, Beijing, China

**Keywords:** *Artemisia argyi*, dominant population, harmful weeds, allelochemicals, caffeic acid, transcriptome, phytohormone

## Abstract

*Artemisia argyi* is widely distributed in Asia, and it often becomes the dominant population in the field because of its strong ecological niche competitiveness. Allelochemicals secreted by plants are generally considered an important reason for their dominance in ecological competition. In this study, the allelochemicals in *A. argyi* were screened by a series of experiments and their mechanisms were explored via transcriptomics. First, the inhibitory effects of *A. argyi* on *Echinochloa crusgalli*, *Setaria viridis*, *Portulaca oleracea* and *Amaranthus retroflexus* were evaluated. Then, we carried out a qualitative and quantitative analysis of the chemical composition of the aqueous extract of *A. argyi* to screen for potential allelochemicals that can inhibit weed growth. Four potential allelochemicals were quantified: neochlorogenic acid (5-CQA), chlorogenic acid (3-CQA), cryptochlorogenic acid (4-CQA), and caffeic acid (CA). Coincidentally, their allelopathic effects on weeds seemed to be identical to their content, in the order CA>4−CQA>5−CQA>3-CQA. These findings suggested that CA might be the main allelopathic compound in the aqueous extract of *A. argyi*. Subsequently, the allelopathic effect and molecular mechanism of CA on *S. viridis* leaves were investigated. The physiological results showed that CA significantly induced reactive oxygen species (ROS) production, led to malondialdehyde (MDA) accumulation, and disrupted enzyme activities (POD, SOD, CAT) in *S. viridis* leaves. Moreover, transcriptome results revealed that CA inhibited *S. viridis* growth by downregulating multiple genes involved in gibberellin (GA) and phytoalexin biosynthesis and Mitogen-activated protein kinase (MAPK) signaling pathways. In addition, differentially expressed genes (DEGs) related to the biosynthesis and signaling pathways of phytohormones were verified by Quantitative Real-Time PCR (RT-qPCR). Taken together, this study may be the first to identify allelochemicals and explore their molecular mechanism about *A. argyi*. Importantly, the ecological advantages of *A. argyi* could be applied to ecological regulation and the development of botanical herbicides.

## Introduction

*Artemisia argyi* is a perennial herb of the *Artemisia* genus in the Compositae family, which is mainly used by moxibustion to prevent and treat multifarious disease at present. *A. argyi* is widely known in Asia and is principally distributed in China, Japan and Korea, indicating strong ecological adaptability (Lan et al., 2020). Interestingly, in previous exploration as fertilizer in the field, we accidentally found that the number of weeds and biodiversity decreased significantly after the treatment of *A. argyi* (Li et al., 2021a). Further experiments confirmed that the extract of *A. argyi* had a great inhibitory effect on the seedling growth of *Brassica pekinensis*, *Lactuca sativa* and *Oryza sativa* in our previous study, among which the aqueous extract was the strongest. These results suggested that *A. argyi* exhibited the strong ecological competitiveness and was an ecologically dominant plant. However, the cause and molecular mechanism of this scientific phenomenon are not clear.

Allelopathy, is a widely existing biological phenomenon that refers to the secondary metabolites secreted by plants or their decomposition products released into the soil and the environment, which affect the growth and development of other plants (Cheng and Cheng, 2015; Cheng et al., 2016). At present, allelopathy is considered an important reason for the dominance of plants in ecological competition, and plants with strong allelopathy are generally the ecological dominant population (Scognamiglio and Schneider, 2020). Because the competition among plants usually inhibits the growth of other individuals by secreting large quantities of allelochemicals and preempt the basic ecological niche of other plants to obtain more space, light, water and nutrients to meet their physiological needs for growth and development (Iqbal et al., 2019). For example, walnut tree secretes walnut quinone to inhibit the growth of surrounding weeds, showing ecological advantages. Additionally, an increasing number of studies have examined the application of allelochemicals in plants to effectively control weed growth in recent years (Iqbal et al., 2016; Ladhari et al., 2018). These plant-derived allelochemicals have been widely studied because of their advantages of environmental friendliness, low toxicity, easy degradation and lack of residue (Ghimire et al., 2020). Thus, it is of great significance to clarify the allelochemicals secreted by *A. argyi* and their molecular mechanism for elucidating its ecological advantages and regulating the ecological balance.

There are many kinds of allelopathic substances and generally constitute phenolic compounds, quinones, coumarins, terpenes, sugars, glycosides, alkaloids, and non-protein amino acids (Iqbal and Fry, 2012). As reported (Liu et al., 2013; Huang et al., 2020), phenolic compounds are considered the major group of plant allelochemicals and could affect a variety of physiological and biochemical processes in plants, including inhibition of cell division, elongation, membrane permeability, enzyme function and activity, hormone expression, plant photosynthesis, and respiration. Notably, studies have shown that phenolic compounds are also the main chemical constituents of *A. argyi*, including flavonoids and phenolic acids (Lan et al., 2020). In our previous study, we speculated that caffeic acid (CA), which is concentrated in the aqueous solution, might be an important allelochemical of *A. argyi*, but this hypothesis remains to be further verified.

**FIGURE 1:**
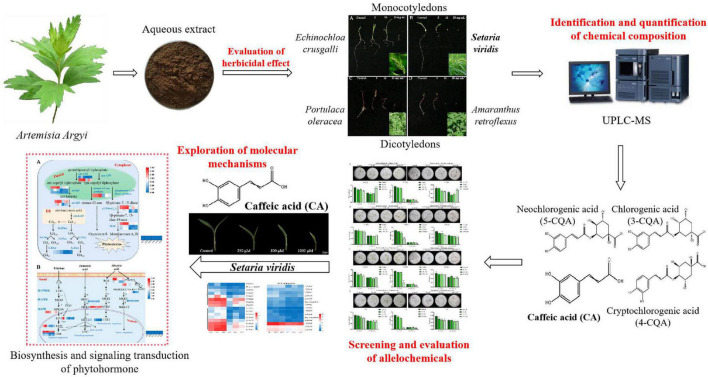
Schematic diagram of screening the allelochemicals and exploring its molecular mechanisms of *A. argyi*.

The monocotyledonous weeds *Setaria viridis* and *Echinochloa crusgalli*, and the dicotyledonous weeds *Portulaca oleracea* and *Amaranthus retroflexus* are common malignant weeds in the field and are widely distributed in temperate, tropical and subtropical regions of the world. Like *A. argyi*, they are also the dominant species in the ecological environment, and they are highly invasive, causing serious environmental pressure to agriculture. In our present study (Figure 1), we first examined the allelopathic inhibitory effect of the aqueous extract of *A. argyi* on four harmful weeds and further verified its role in inhibiting weed growth. As a result, we identified the broad-spectrum characteristics and differences in weed inhibition of *A. argyi*. Then, the chemical composition in the *A. argyi* aqueous solution was identified and quantified to determine the potential allelochemicals that can inhibit weeds. Meanwhile, we detected the leaf morphology and physiological traits, including reactive oxygen species (ROS) production, malondialdehyde (MDA) content, and antioxidant enzyme activities of weeds subjected to allelochemical treatments. Furthermore, transcriptomic analysis was conducted to compare the differences in gene expression between the control group and allelochemical group to screen out the key molecular mechanisms of allelochemicals in *A. argyi*. Finally, the differentially expressed genes (DEGs) in key pathways were verified by Quantitative Real-Time PCR (RT-qPCR). In brief, this study will provide a powerful scientific support for revealing *A. argyi* as an ecological dominant plant. Moreover, the population ecological advantage of *A. argyi* is an important guarantee for ecological regulation and the development of botanical herbicides.

## Materials and Methods

### Plant Materials and Chemicals

The *A. argyi* was collected from Qichun County, Hubei Province, China. Weed seeds are *E. crusgalli*, *S. viridis*, *P. oleracea*, and *A. retroflexus*, purchased in agricultural product market (Wuhan, China). Neochlorogenic acid (5-CQA, 99.6%), chlorogenic acid (3-CQA, 99.5%), cryptochlorogenic acid (4-CQA, 98.7%), and caffeic acid (CA, 99.7%) were provided by Chengdu Desite Bio-Technology Co., Ltd. (Chengdu, China). The HPLC grade acetonitrile, methanol, and formic acid (FA) were purchased from Merck (Darmstadt, Germany). Ultrapure water was prepared using a Milli-Q purification system (Millipore Laboratory, Bedford, MA, United States). Other chemicals or solvents were of analytical grade and used without further purification.

### Aqueous Extract Solution Preparation

The dried *A. argyi* was pulverized and sieved, and was ultrasonically extracted with 5 times of pure water at room temperature for 2 times, 60 min each time. Then the filtrate was concentrated and freeze-dried to obtain the aqueous extract of *A. argyi*. After dissolving the extract powder with water, a stock solution (20 mg⋅mL^–1^) was obtained. Neochlorogenic acid (5-CQA), chlorogenic acid (3-CQA), cryptochlorogenic acid (4-CQA), and caffeic acid (CA) were also ultrasonically dissolved in water to prepare the stock solution at room temperature (1,000 μM). The all aqueous solutions were filtered through at 0.22 μm Millipore membrane to remove microorganisms, and then stored at 4^°^C until use.

### Chemical Composition Analysis

The chemical composition identification of *A. argyi* was performed by an Waters Acquity I-Class ultra-performance liquid chromatography combined with a Zevo G2-XS quadrupole time-of-fight mass spectrometer (Waters, Milford, MA, United States). Waters Acquity UPLC HSS T3 column (100 mm × 2.1 mm, 1.8 μm) was used at 40^°^C for a gradient mobile phase composed of A (0.1% FA in water) and B (acetonitrile), and the flow rate was 0.5 ml⋅min^–1^. The gradient elution program was 0∼0.3 min: 2–5% B, 0.3∼1.0 min: 5% B, 1.0∼10.0 min: 5–28% B, 10.0∼16.0 min: 28–45% B, 16.0∼19.0 min: 45–70% B, 19.0∼20.5 min: 70–98% B, 20.5∼24.0 min, 98% B, 24.0∼24.2 min: 98–2% B, and 24.2∼27.0 min: 2% B. The mass spectrometer was operated in MS^E^ mode. The compounds were fragmented, and their scan product ion (m/z 50–1,200) were recorded in negative ionization mode. Meanwhile, the external standard method was used to determine the content of the main allelochemicals (5-CQA, 3-CQA, 4-CQA, and CA) in the aqueous extract of *A. argyi*.

The total phenolic content (TPC) and total flavonoid content (TFC) of the aqueous extract were measured following previously reported procedures (Tian et al., 2015; Huang et al., 2021). The used standards were gallic acid or rutin (20–100 μg⋅mL^–1^), respectively. The TPC and TFC were expressed as milligram of gallic acid (mg GAE/g Fr.) or rutin (mg RuE/g Fr.) equivalent per gram of fraction, respectively. All measurements were performed in triplicate to calculate the average values.

### Experimental Design

The disinfection and culture of *E. crusgalli, S. viridis, P. oleracea*, and *A. retroflexus* are carried out according to our previous research (Li et al., 2021a). Twenty seeds from the above tested species were equidistantly placed in petri dish (diameter = 90 mm) with two layers of filter paper. Then we treated them with 8 mL of an aqueous solution gradient (5, 10, and 20 mg⋅mL^–1^), and the concentration gradients of 5-CQA, 3-CQA, 4-CQA, and CA were 10, 100, and 1,000 μM. The control groups were treated with the same volume of pure water, and all treatments were performed in triplicate. Subsequently, 2 mL of corresponding aqueous solution was added at the same time every other day to replenish the solution that had been consumed. The dishes were placed in a growth chamber at a constant temperature of 28 ± 1°C, 85% humidity, and a controlled 12 h light/12 h dark cycle. After 7 days of treatment, stem length, root length, and biomass of each group were measured.

### Measurement of Reactive Oxygen Species Production, Malondialdehyde Content, and Antioxidant Enzyme Activities of Leaves

Reactive oxygen species in leaves were detected by chemical fluorescence assay. Briefly, 600 μL phosphate-buffered saline (PBS, 10 mM, PH 7.4) was added to the 50 mg leaves of the treatment and control groups for homogenization, and the supernatant was taken after centrifugation. Next, 150 μL supernatant was mixed with 50 μL DCFH-DA probe (10 mM) and reacted for 30 min in the dark. After that, the mixture was placed under the fluorescence inverted microscope (Olympus, Japan) to observe the green fluorescence.

Malondialdehyde content was measured using the thiobarbituric acid colorimetric method. Peroxidase (POD), catalase (CAT), and superoxide dismutase (SOD) activities were measured by guaiacol, ammonium molybdate, and WST-1 methods, respectively. The specific operation was carried out according to the kit instructions. The kits were purchased from Nanjing Jiancheng Bioengineering Institute (Nanjing, China). The above experiment was repeated for three times.

### RNA Isolation and RNA-Seq

*Setaria viridis* was selected as the test plant, of which the leaves treated with 1,000 μM CA were selected for RNA isolation. The leaves were also treated for 7 days with three independent biological replicates. According to the manual instructions, the total RNA was extracted from *S. viridis* by using CTAB-PBIOZOL methods (Liu et al., 2020). After that, selecting RNA with well integrity (RIN ≥ 6.0) to library preparation. Firstly, rRNA was removed with RNase H, followed by digestion with DNase I, which was used to remove both double and single-stranded DNA. Then, the purified mRNA were cleaved into short fragments at appropriate temperature by breaking reagent. The first cDNA strand was synthesized using the mRNA fragments, random primers and reverse transcriptase, followed by the second cDNA strand. Next, the cDNA fragments were end-repaired by adding “A” base and ligating adapters, and PCR was used for amplification to form the final cDNA library. Last, RNA library was sequenced on BGISEQ500 platform in Beijing Genomics Institute (BGI, Shenzhen, China).

### Sequencing Analysis and Differentially Expressed Genes Analysis

The sequencing data was filtered with SOAPnuke (v1.5.2), and the clean reads were mapped to the genome reference of *S. viridis* (*Setaria viridis* v2.0) using HISAT2 (v2.0.4). The obtained genes from the previous step were compared and quantitatively analyzed using Bowtie2 (v2.2.5) and RSEM (v1.2.12). The fold change (FC) between the treatment and control groups was calculated for each transcript. In order to screen the key genes of CA inhibiting the growth of weeds, DEGs were cut to adjust *P*-value < 0.05, log_2_| FC| ≥ 1, and heatmap in at least one total comparison. The Gene Ontology (GO)^1^ analysis and Kyoto Encyclopedia of Genes and Genomes (KEGG)^2^ pathway enrichment analysis were performed to find the leading pathways.

### Validation of Differentially Expressed Genes by Quantitative Real-Time PCR

For DEGs validation, *S. viridis* samples were prepared with the concentration gradients of CA (250, 500, and 1,000 μM) for 7 d. The total RNA of leaves was isolated using TRIzol (Tiangen Bio Co., Ltd., Beijing, China), and cDNA was synthesized using the MLV reverse transcriptase (Promega Corporation, United States). RT-qPCR was conducted using RealUniversal Color PreMix (SYBR Green) according to the manufacturer’ s protocol (Tiangen, Beijing, China). The expression level of genes was normalized with IF4A (Saha et al., 2016). The primer sequences used for the RT-qPCR analysis are provided in Supplementary Table 1.

### Statistical Analysis

Data were recorded and analyzed using GraphPad Prism 8.0 and SPSS 24.0 software. One-way analyses of variance (ANOVA) followed by multiple comparison tests of means (Tukey’s test) was used to compare the difference between the experimental and control groups, *P*-value < 0.05 was considered as significant differences. All experiments were performed in triplicate.

## Results

### Effect of *Artemisia argyi* on the Growth of Harmful Weeds

**FIGURE 2:**
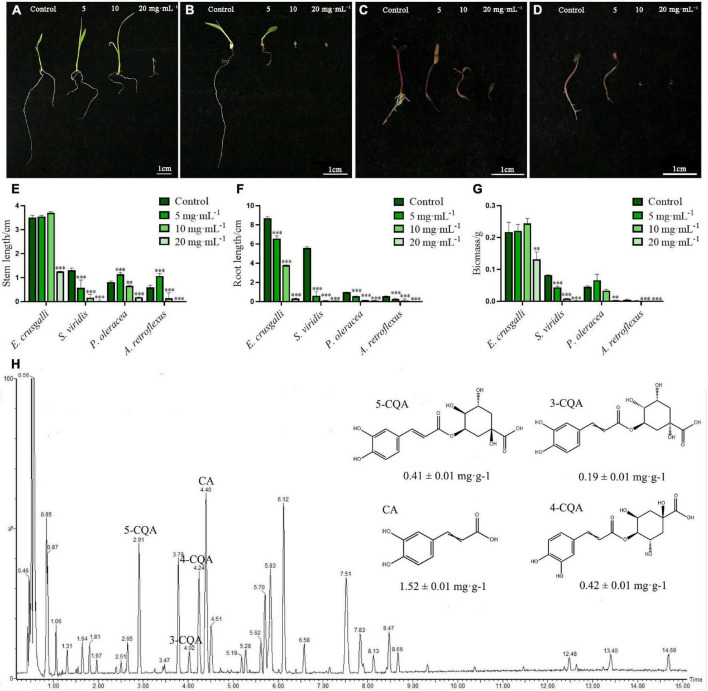
Suppression effects on the four harmful weeds and identification of chemical constituents of the *A. argyi* aqueous extract. Panels **(A–D)** present the growth status of *E. crusgalli*, *S. viridis*, *P. oleracea* and *A. retroflexus* with the treatment of *A. argyi*, respectively; panels **(E–G)** present the suppression effects on the stem length, root length, and biomass of four harmful weeds, respectively; panel **(H)** presents the UPLC-MS diagram of the *A. argyi* aqueous extract in negative ionization mode; * indicates *p* < 0.05, ^**^ indicates *p* < 0.01, and ^***^ indicates *p* < 0.001 relative to the control by ANOVA. The data are presented as the mean ± standard deviation (*n* = 3).

To investigate the effects of the aqueous extract of *A. argyi* on the growth of different harmful weeds, we selected monocotyledonous *E. crusgalli* and *S. viridis*, and dicotyledonous *P. oleracea* and *A. retroflexus* to observe the stem length, root length, and biomass. The results showed that growth of the four weeds was suppressed by the aqueous extract in a concentration-dependent and hormesis manner (Figure 2). In this study, the stem length and biomass of *E. crusgalli*, *P. oleracea*, and *A. retroflexus* were slightly increased at *A. argyi* aqueous extract concentrations of 5 and/or 10 mg⋅mL^–1^, but were significantly inhibited at a concentration of 20 mg⋅mL^–1^ (*P* < 0.001). The extract showed a typical allelopathic effect of promotion at low concentrations and inhibition at high concentrations. Root length was significantly inhibited in a concentration-dependent manner (*P* < 0.001). For *S. viridis*, the three growth indices showed a significant reduction even at a concentration of 5 mg⋅mL^–1^. When the concentration was higher than 5 mg⋅mL^–1^, most of the aboveground and belowground parts did not grow. In brief, the *A. argyi* aqueous extract could inhibit the growth of four harmful weeds to different degrees. Among them, *S. viridis* was the most sensitive to *A. argyi* aqueous extract.

### Chemical Composition Analysis of the *Artemisia argyi* Aqueous Extract

To better understand the associated allelochemicals and the ecological advantages of *A. argyi*, it is crucial to identify the allelochemicals. We used UPLC/Q-TOF-MS to analyze the chemical constituents of the aqueous extract of *A. argyi* (Figure 2H). Indeed, 14 main peaks scanned in the *A. argyi* aqueous extract were related to known molecules (Supplementary Table 2), including 13 phenolic compounds and one organic acid (peak 7). In particular, there were 9 phenolic acids in 13 phenolic compounds, suggesting that phenolic acids were the main active constituents of the aqueous extract of *A. argyi*. Furthermore, the main phenolic acids were quantitatively analyzed in *A. argyi* aqueous extract. Obviously, CA had the highest contents (1.52 ± 0.01 mg⋅g^–1^) in the aqueous extract of *A. argyi*, which was more than 3 times that of the other compounds, followed by 4-CQA, 5-CQA, and 3-CQA in succession (Supplementary Table 3). In addition, the TPC and TFC of *A. argyi* aqueous extract were also measured and are presented in Supplementary Table 3. The results indicated that the TPC (78.40 ± 1.28 mg GAE/g Fr.) was more than 2 times the TFC (30.19 ± 0.19 mg RuE/g Fr.), which further confirmed that phenolic acids were the main components. Therefore, it is necessary to evaluate the allelopathic potential of phenolic acids in the *A. argyi* aqueous extract to provide scientific support for the ecological advantage of *A. argyi*.

### Allelopathic Effects of the Four Compounds

**FIGURE 3:**
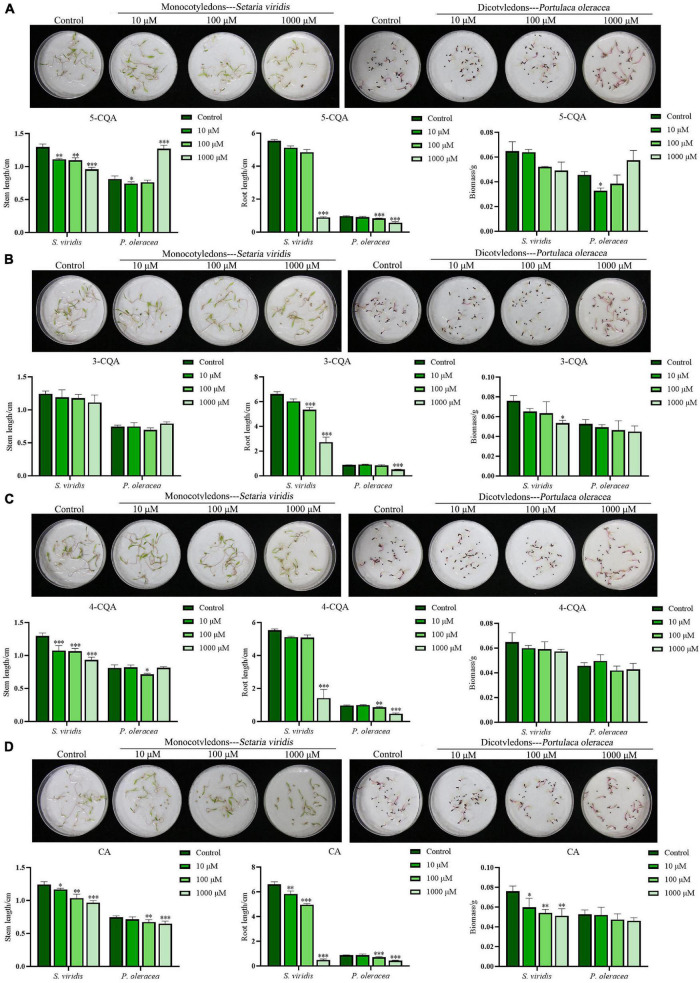
Suppression effects of 5-CQA, 3-CQA, 4-CQA, and CA on *S. viridis*, and *P. oleracea.* Panels **(A–D)** present the suppression effects on the stem length, root length, and biomass of *S. viridis*, and *P. oleracea* by 5-CQA, 3-CQA, 4-CQA, and CA, respectively; * indicates *p* < 0.05, ^**^ indicates *p* < 0.01, and ^***^ indicates *p* < 0.001 relative to the control by ANOVA. The data are presented as the mean ± standard deviation (*n* = 3).

Based on the quantitative analysis, 5-CQA, 3-CQA, 4-CQA, and CA, four phenolic acids, were considered potential allelochemicals in *A. argyi*. In this experiment, *S. viridis* and *P. oleracea* were selected to evaluate the allelopathic effects of the four compounds, as in the above study of the aqueous extract of *A. argyi*. As shown in Figure 3, the sensitivity of *S. viridis* to the four compounds was higher than that of *P. oleracea*. 5-CQA and 4-CQA could significantly inhibit the stem length of *S. viridis* at concentrations ranging from 10 to 1,000 μM (*P* < 0.01), but less inhibition of *P. oleracea* was observed for these treatment groups, and 5-CQA even significantly increased the stem length of *P. oleracea* at 1,000 μM (*P* < 0.0001) (Figures 3A,C). Regarding root length, the two compounds had no significant suppressive effect on the root length of *S. viridis at* 10 and 100 μM but could significantly inhibit root length at high concentrations (1,000 μM). Simultaneously, 5-CQA and 4-CQA suppressed the root length of *P. oleracea* and had significant effects at 100 μM (Figures 3A,C). 3-CQA dramatically decreased the root length of the two weeds at higher concentrations (100 or 1,000 μM) while exerting a less suppressive effect on stem length (Figure 3B). Notably, CA had favorable dose-dependent effects on the growth of the two weeds for both stem length and root length (Figure 3D). Moreover, the suppressive effect of CA was more remarkable than that of the other three compounds. In addition, only CA markedly suppressed the biomass of *S. viridis* and *P. oleracea* (Figure 3D). In summary, the allelopathic inhibitory effects of the four compounds exhibited the following order: CA>4−CQA>5−CQA>3-CQA. The above quantification of CA shows that it may be an important weapon for *A. argyi* to inhibit the growth of other plants and show ecological advantage.

### Caffeic Acid Caused Oxidative Damage and Regulated the Antioxidative Enzyme Activities of *Setaria viridis* Leaves

**FIGURE 4:**
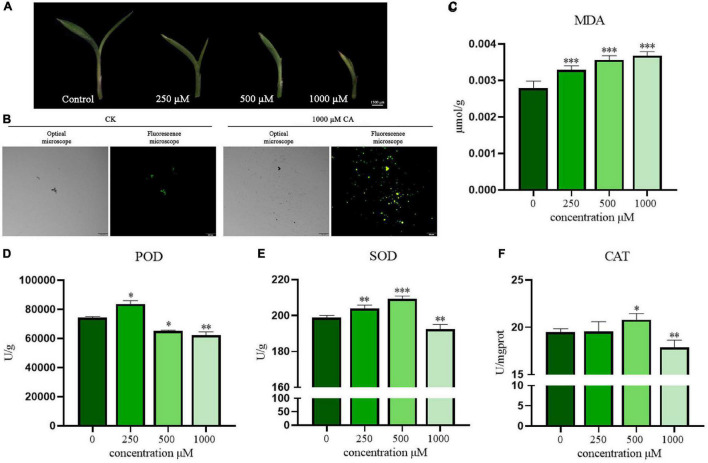
Suppression effects of CA on leaves morphology, ROS production, MDA content, and antioxidative enzyme activities of *S. viridis*. Panel **(A)** represents leaves morphology of *S. viridis*; panels **(B,C)** represent ROS production and MDA content after CA treatment, respectively; panels **(D–F)** represent the activities of POD, SOD, and CAT after CA treatment, respectively; * indicates *p* < 0.05, ^**^ indicates *p* < 0.01, and ^***^ indicates *p* < 0.001 relative to the control by ANOVA. The data are presented as the mean ± standard deviation (*n* = 3).

As shown in Figure 4A, the effect of a CA concentration gradient (250, 500, and 1,000 μM) on the appearance and morphology of *S. viridis* leaves was observed by stereoscopic microscope (MZ101, China). CA had a significant inhibitory effect on *S. viridis* leaves in a concentration-dependent manner. Compared with the control group, CA showed great suppression of the growth and development of stems and leaves at the concentration of 250 μM. With increasing CA concentration, the leaves curled gradually, and the growth of the middle leaflet was seriously hindered. Finally, the leaves curled completely at a concentration of 1,000 μM, which was accompanied by gradual yellowing and drying. ROS are thought to heavily accumulate under abiotic stress, accelerating plant senescence and death (Espinosa-Vellarino et al., 2020). Subsequently, the oxidative stress induced by CA was investigated. ROS production in *S. viridis* leaves was evaluated by a chemical fluorescence assay (Figure 4B). The green fluorescence in *S. viridis* leaves treated with CA was obviously stronger than that in the control group (CK). This result indicated that CA markedly increased the accumulation of ROS and stimulated the oxidative stress response of *S. viridis*. Meanwhile, the excessive accumulation of lipid peroxidation products was also observed (Figure 4C). Specifically, CA induced the accumulation of MDA, and MDA content in *S. viridis* leaves increased with increasing CA concentration (250–1,000 μM). Therefore, these results indicated that CA could induce an increase in ROS production in *S. viridis* leading to lipid peroxidation, which finally disrupted cell functions and enzyme activities.

The plant antioxidant enzyme system, including POD, SOD, and CAT, is closely related to the maintenance of ROS homeostasis. These enzymes can protect cells from damage by scavenging free radicals and attenuating their toxic effects (Hu et al., 2020). In this study, the overall trend of changes in POD, SOD, and CAT activities showed the allelopathic effect of promotion at low concentrations but remarkable suppression at high concentrations. Briefly, POD activity increased significantly at 250 μM and decreased in a concentration-dependent manner beginning at 500 μM (*P* < 0.05) (Figure 4D). In contrast, SOD and CAT activities increased even at 500 μM but significantly decreased at 1,000 μM (*P* < 0.01) (Figures 4E,F). These results suggest that cells can activate their own antioxidant defense system over time under low-pressure conditions. Once the stress exceeds their tolerance, oxidative damage will become very serious, eventually inhibiting the normal growth of plants. This shows that when the allelochemicals secreted by *A. argyi* accumulate to a sufficient amount, it will inhibit the growth of the surrounding plants and become the ecologically dominant population.

### Differential Gene Expression Analysis

The above results showed that CA obviously inhibited the growth of *S. viridis* leaves, but the molecular mechanism is unknown. Therefore, we selected the control group (CK) and CA treatment group (1,000 μM) for transcriptomic analysis to identify candidate genes and pathways involved in the suppressive effect of CA on *S. viridis*. First, six libraries were created and sequenced from three biological replicates for the CK and CA groups of *S. viridis* leaves based on the DNBSEQ platform. After quality control and filtering for each sample, more than 42.02 million (6.30 Gb) clean reads were obtained. The proportion of nucleobases with mass values greater than 30 in clean reads was more than 92.15%, and 92.19∼93.34% of clean reads were mapped to the reference genome (Supplementary Table 4).

**FIGURE 5:**
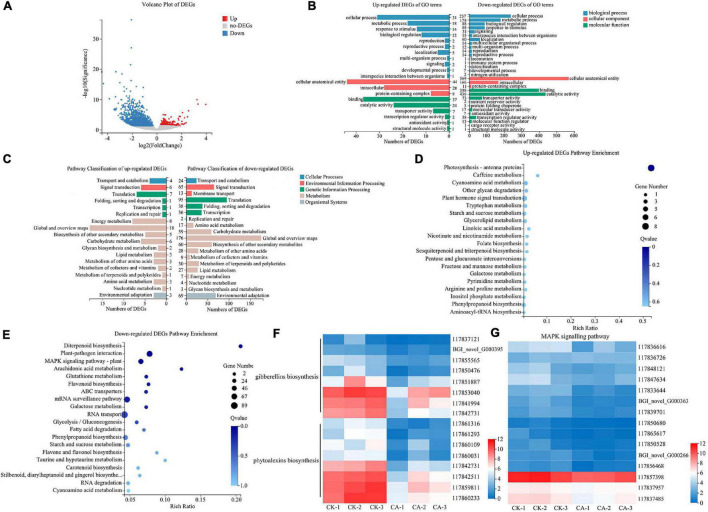
RNA-Seq analysis of *S. viridis* under CA treatment. **(A)** The volcano plot of DEGs; **(B)** the GO terms classification analysis of DEGs; **(C)** the KEGG pathway classification analysis of DEGs; **(D)** top 20 KEGG pathway enrichment analysis of up-regulated DEGs; **(E)** top 20 KEGG pathway enrichment analysis of down-regulated DEGs; **(F)** the expression of key DEGs involved in the biosynthesis of gibberellins (GAs) and phytoalexins; **(G)** the expression of key DEGs involved in the MAPK signaling pathway; Blue and red bands were used to represent low and high gene expression levels, respectively.

Analysis of DEGs between the CK and CA groups was conducted using DEseq2 after comparisons of the fragments per kilobase of transcript length per million mapped reads (FPKM) values. Statistical analysis showed that 1,747 DEGs were detected, including 367 upregulated genes and 1,380 downregulated genes. However, we focused on the evaluation of genes with a FC greater than 2 (log_2_| FC| ≥ 1) to further screen key candidate DEGs. In total, only 81 genes were upregulated, but 960 genes were downregulated (Figure 5A). This result indicated that CA suppressed the growth of *S. viridis* mainly by downregulation of related genes.

### Functional Classification of Differentially Expressed Genes by Enrichment Analysis

To further identify the major functional categories of DEGs in the CK and CA groups, GO enrichment analysis was carried out. Upregulated and downregulated DEGs were categorized into 20 and 30 GO terms, respectively (Figure 5B). In the biological process category, the largest class was cellular process in both up-regulated and down-regulated DEGs, with 31 and 237 enriched DEGs, respectively. Among the cellular component category, 44 upregulated and 569 downregulated DEGs were enriched in cellular anatomical entity. For the molecular function category, the largest classes were binding in the upregulated DEGs and catalytic activity in the downregulated DEGs, with 37 and 436 DEGs, respectively.

We also performed KEGG enrichment analysis of the DEGs to identify the main pathways active in the allelopathic effects of CA on *S. viridis* growth. The results showed that the DEGs of upregulation and downregulation were enriched in 18 and 19 pathways, respectively (Figure 5C). Both upregulated and downregulated DEGs were mainly enriched in “Metabolism” and “Genetic information processing,” specifically related to “Global and overview maps,” “Carbohydrate metabolism,” “Biosynthesis of other secondary metabolites,” and “Translation.” In detail, “Photosynthesis-antenna proteins” was the only significantly enriched pathway in upregulated DEGs (8 unigenes) (Figure 5D). We suggest that the reason for this phenomenon was the stress response after *S. viridis* was exposed to CA. For downregulated DEGs, “Diterpenoid biosynthesis” (19 unigenes), “Plant-pathogen interaction” (69 unigenes), and “Mitogen-Activated Protein Kinase (MAPK) signaling pathway-plant” (49 unigenes) were the most significantly enriched pathways (*Q*-value < 0.05) (Figure 5E). Specifically, 15 unigenes were involved in the biosynthesis of gibberellins (GAs) and phytoalexins in the diterpenoid biosynthesis pathway (Figure 5F), and 15 unigenes were relevant to MAPK-mediated signal transduction pathways of plant hormones (Figure 5G), such as ethylene (ET), jasmonic acid (JA), and abscisic acid (ABA). These results revealed that CA inhibited weed growth via multiple targets and pathways, especially by inhibiting the biosynthesis and signal transduction of plant hormones.

### Differentially Expressed Genes Involved in Diterpenoid Biosynthesis

**FIGURE 6:**
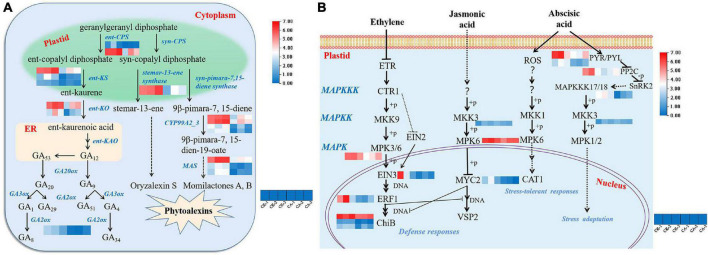
Gene expression in diterpenoid biosynthesis and MAPK signaling pathways under CA treatment. **(A)** The biosynthesis of gibberellins (GAs) and phytoalexins; **(B)** the MAPK-mediated signal transduction pathways of plant hormones. Blue and red bands were used to represent low and high gene expression levels, respectively.

The DEGs involved in diterpenoid biosynthesis (ko00904), especially those involved in the biosynthesis of GAs, were further investigated. Under CA stress, most pathways and enzymes involved in GA biosynthesis were differentially altered. As shown in Figure 6A, enzymes regulating the GA pathway, including ent-copalyl diphosphate synthase (ent-CPS, EC-5.5.1.13), ent-kaurene synthase (ent-KS, EC-4.2.3.19), ent-kaurene oxidase (ent-KO, EC-1.14.14.86), and GA 2beta-dioxygenase (GA2ox, EC-1.14.11.13), were all significantly inhibited in *S. viridis* by CA treatment. A total of 8 genes were significantly downregulated.

In addition, the pathway by which the diterpene precursor GGPP (geranylgeranyl diphosphate) synthesizes stemar-13-ene and 9β-pimara-7, 15-diene catalyzed by syn-CPS was suppressed. These are considered to be important intermediates in the synthesis of the phytoalexins oryzalexin S, momilactones A and B (Nemoto et al., 2004). One gene encoding stemar-13-ene synthase, 4 genes encoding CYP99A2/3 and 3 MAS genes were downregulated (Figure 6A). This result showed that CA inhibited weed growth via suppression of GA and phytoalexin biosynthesis pathways.

### Differentially Expressed Genes Involved in the Mitogen-Activated Protein Kinase Signaling Pathway

Mitogen-activated protein kinase cascades, which are evolutionarily conserved signaling modules in all eukaryotes, serve to transduce extracellular signals to the nucleus or cytoplasm to produce appropriate cellular responses, including cell division, differentiation, programmed cell death, and adaptation to various stresses (Zhang et al., 2018). The MAPK cascade signal transduction pathway (ko04016) is modulated by stress-related phytohormones. In this study, 15 hormone signal transduction-related genes involved in MAPK cascade modulation were significantly downregulated (Figure 6B). *MAPKKK17/18* (MAP2K kinase), *MKK3* (MAPK kinase), *MPK3* and *MPK6* which are involved in ET, JA, and ABA signal transduction were significantly inhibited. Furthermore, the expression of most downstream products of ET and JA phosphorylated by the MAPK cascade, including *EIN3*, *ERF1*, *ChiB*, and *MYC2*, was downregulated. In contrast, the major upstream genes involved in ABA signal transduction were expressed at low levels, including *PYR/PYL* and *PP2C*. In summary, suppression of the plant hormone signaling pathway is a key mechanism by which CA inhibits weed growth.

### Quantitative Real-Time PCR Validation of Differentially Expressed Genes

**FIGURE 7:**
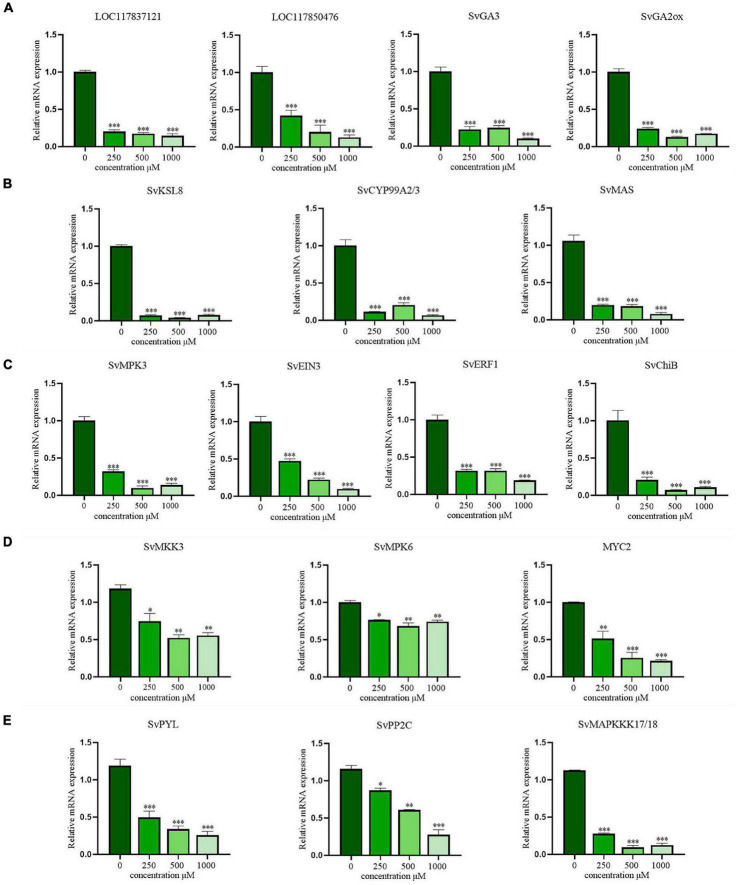
The verification of RT-qPCR in gibberellins (GAs) biosynthesis, phytoalexins biosynthesis and MAPK signaling pathways. **(A)** The biosynthesis of gibberellins (GAs); **(B)** the biosynthesis of phytoalexins; **(C)** the signal transduction pathway of ethylene (ET); **(D)** the signal transduction pathway of jasmonic acid (JA); **(E)** the signal transduction pathway of abscisic acid (ABA). The data are presented as the mean ± standard deviation (*n* = 3). * indicates *p* < 0.05, ** indicates *p* < 0.01, and *** indicates *p* < 0.001 relative to the control by ANOVA.

The expression of key DEGs involved in the allelopathy of CA was validated by RT-qPCR. First, four DEGs involved in the biosynthesis of GAs were selected for qRT-PCR analysis (Figure 7A). *LOC117837121* (encoding ent-CPS), *LOC117850476* (encoding ent-KS), *SvGA3* (encoding ent-KO), and *SvGA2ox* (encoding GA 2beta-dioxygenase) were all significantly downregulated in a concentration-dependent manner (*P* < 0.001), even at low concentrations. Additionally, *SvKSL8* (encoding stemar-13-ene synthase), *SvCYP99A2/3* (encoding 9beta-pimara-7,15-diene oxidase), and *SvMAS* (encoding momilactone-A synthase), three DEGs related to phytoalexin synthesis were also verified and exhibited concentration-dependent inhibition (Figure 7B). These results were consistent with the RNA-seq data, suggesting that CA could indeed exert allelopathic effects by inhibiting the biosynthesis of GA and phytoalexin.

Moreover, the key DEGs in plant hormone signal transduction pathway mediated by the MAPK cascade were also analyzed. For ET, DEGs regulating *SvMPK3* and its downstream transcription factors (*SvEIN3*, *SvERF1*, and *SvChiB*) were significantly downregulated (Figure 7C). Similarly, the expression tendencies of genes and transcription factors involved in JA signaling pathways, including *SvMKK3*, *SvMPK6*, and *SvMYC2*, were consistent with the transcriptome results (Figure 7D). After CA treatment, the major DEGs involved in ABA signal transduction, including *SvPYR/PYL* and *SvPP2C*, were still expressed at low levels (Figure 7E). Therefore, the inhibition of hormone signal transduction in *S. viridis* leaves is also an important pathway of the allelopathic mechanism of CA.

## Discussion

In recent years, with the proliferation of weeds and the abuse of chemical herbicides, an increasing number of allelopathic plants have been found to inhibit the growth of harmful weeds. For instance, *Ficus carica* suppresses *Peganum harmala* and *Silybum marianum* (Ladhari et al., 2020), allelopathic rice significantly inhibits *E. crusgalli* (Li et al., 2020), and *Miscanthus sacchariflorus* can also suppress weed growth (Ghimire et al., 2020). In our previous study, field observation and laboratory experiments have confirmed that *A. argyi* is an ecologically dominant population and can also be used for weed control. Based on our previous discovery, we further evaluated the inhibitory effect of the *A. argyi* aqueous extract on four harmful weeds, namely, *E. crusgalli*, *S. viridis, P. oleracea*, and *A. retroflexus*. The results showed that *A. argyi* had a significant allelopathic effect on both monocotyledonous and dicotyledonous weeds, exhibiting a broad spectrum of herbicidal activity. Then, based on a conjoint analysis of chemical composition and biological activity, CA was found to be the key allelochemical in *A. argyi*. Moreover, physiological and transcription analyses revealed the allelopathic mechanism by which CA inhibits weed growth. Finally, combined with RT-qPCR validation, suppression of GA and phytoalexin biosynthesis and the MAPK signaling pathways were considered key mechanisms of the effect of CA. These results reasonably revealed that the strong niche of *A. argyi* in the wild might arise from the allelochemicals released by them affecting the hormone metabolism of other plants. Importantly, this study also provided powerful scientific support for the development of environmentally friendly and resource-saving herbicides.

Phenolic compounds are a class of important and common plant allelochemicals in ecosystems, and phenolic acids are indispensable among them (Macías et al., 2019). p-Hydroxybenzoic acid inhibits root growth by regulating ROS accumulation in cucumber, resulting in a continuous obstacle for crop growth (Huang et al., 2020). Phenolic acids including vanillic acid, ferulic acid, p-coumaric acid, and syringic acid were also found to inhibit the growth and development of other plants (Huang et al., 2017). In this study, 14 peaks were detected in the *A. argyi* aqueous extract by UPLC-MS (Figure 2); of which, 9 peaks were proposed as phenolic acids based on the retention times, experimental molecular ions ([M-H]^–^), major MS fragment, and published MS data (Han et al., 2017). This result indicated that phenolic acids present in the *A. argyi* aqueous extract might be a potential source of bioherbicides. Previous studies have confirmed that phenolic acids and flavonoids are the main secondary metabolites in *A. argyi* (Lan et al., 2020). Whereas, experimental results suggested that the TPC in the *A. argyi* aqueous extract was more than twice the TFC (Supplementary Table 3), further showing that phenolic acids play a key role in the allelopathic effect of *A. argyi*. Quantification of the substances of the *A. argyi* aqueous extract showed that four phenolic acids, 5-CQA, 3-CQA, 4-CQA, and CA, were the most abundant compounds (Supplementary Table 3). Interestingly, their allelopathic effects on weeds seemed to be identical to their content, exhibiting the following order: CA>4−CQA>5−CQA>3-CQA (Figure 3). Notably, 5-CQA, 3-CQA, and 4-CQA are all condensed by CA and quinic acid at different binding sites and produced by plants through the shikimic acid pathway during aerobic respiration (Chang et al., 2021). The differences in biological activity of these isomeric compounds could be caused by the presence of different binding sites. In brief, CA not only had the highest content in *A. argyi* aqueous extract but also exhibited stronger allelopathy at the same concentration, which might be an important weapon for *A. argyi* to inhibit the growth of other plants. Furthermore, as an allelochemical, CA is characterized by its environmental friendliness, easy accessibility, and low price, suggesting that it could be expected to be developed into a plant herbicide. Importantly, this was the first study to quantitatively analyze and evaluate allelochemicals in *A. argyi*, and the allelopathic effects of 5-CQA and 4-CQA were also reported for the first time.

*Setaria viridis* is not only a malignant weed in the field, but also an ideal C4 model plant for the study of abiotic stress because of its small genome, short life cycle and simple growth requirements (Nguyen et al., 2020). In this study, we found that CA could inhibit the growth of aboveground parts of *S. viridis* (Figure 4). *S. viridis* was the weed most sensitive to CA treatment. With increasing concentration, the aboveground parts of *S. viridis* gradually curled and dried, indicating that CA seriously affected its life-sustaining activities (Figure 4A). Many plants are inherently resistant to specific plant toxins or have antioxidant defense mechanisms against allelochemicals produced by other species (Shitan, 2016). ROS are considered to be the most reliable indicator of oxidative stress in plants (Espinosa-Vellarino et al., 2020). ROS levels increased sharply under CA stress, indicating that CA disrupted the ROS homeostasis in *S. viridis* and caused oxidative damage (Figure 4B). Lipid peroxidation is considered the first event mediating broad-spectrum pesticide toxicity (Li et al., 2021b), and the MDA content of the product of lipid peroxidation gradually increased in our study (Figure 4C). Oxidative damage to plants caused by stress usually responds to changes in the activities of some antioxidant enzymes (i.e., POD, SOD, and CAT) (Hu et al., 2020). In the CA treatment group, the activities of antioxidant enzymes were promoted at low concentrations and significantly inhibited at high concentrations of CA (Figures 4D–F). These results showed that *S. viridis* effectively activated its antioxidant defense system to adapt to stress from low concentrations of CA. Conversely, oxidative damage was intensified at high concentrations, which led to ROS production and hindered growth and development. Briefly, the functional imbalance of the antioxidant system in *S. viridis* might be an inhibitory mechanism of CA allelopathy.

Although published studies are available regarding the physiological effects of CA on other plants, but they mainly focus on photosynthesis (Barkosky et al., 2000), protein synthesis (Batish et al., 2008) and element absorption (Glass, 1975). However, little or no information is available about the effects of CA on the synthesis and biological function of hormones. In this study, RNA-seq was applied to analyze the DEGs between the CA treatment and control groups (Zhang et al., 2019; Li et al., 2021a). GO and KEGG enrichment analyses showed that CA could weed growth via multiple targets and pathways (Figure 5). Nevertheless, the pathways related to diterpenoid biosynthesis and MAPK signal transduction in *S. viridis* leaves were significantly inhibited, especially the biosynthesis (GA) and signal transduction (ET, JA, and ABA) of plant hormones (Figure 6). Therefore, this study will provide insight into the molecular mechanism of CA allelopathy.

Fifteen DEGs involved in diterpenoid biosynthesis were significantly downregulated (Figure 6A). Whereas, GAs are not only a large group of diterpenoid carboxylic acids but also important plant hormones. They have been found to promote the growth of higher plants, especially the elongation of stems (Sponsel, 2016). In recent years, knowledge of GA biosynthesis has progressed rapidly. As diterpenoids, GAs are formed from GGPP, which is cyclized in two steps to the tetracyclic hydrocarbon precursor ent-kaurene via ent-copalyl diphosphate in plastids (Hedden, 2020). The two steps are catalyzed by the diterpenoid synthases ent-CPS and ent-KS, respectively. The conversion of ent-kaurene to GA_12_, the first C_20_-GA in the biosynthetic pathway, is catalyzed by two cytochrome P450 monooxygenases, ent-KO and ent-KAO. In our study, *LOC117837121*, *LOC117850476*, and *SvGA3*, which encode ent-CPS, ent-KS, and ent-KO, respectively, were significantly downregulated (*P* < 0.001) (Figure 7). Severe obstruction of GA_12_ biosynthesis led to a decrease in bioactive GAs (GA_1_, GA_4_) (Martínez-Bello et al., 2015). This result was consistent with the obvious shortening of stem length and the leaves died under CA stress. Transcriptomic analysis of emmer wheat suggests that drought stress downregulates *GA2ox*, which may thereby cause an increase in bioactive GAs (Krugman et al., 2011). Down-regulation of *SvGA2ox* was also detected in our study, and we propose that it was a stress response caused by plants attempting to reduce the inactivation of bioactive GAs. In addition, phytoalexin-mediated biosynthesis of diterpenoids was also inhibited. When plants are attacked by pathogenic microorganisms, they respond by a variety of defense reactions, including the production of phytoalexins, which are low-molecular-weight compounds serving as plant antibiotics (Kanfra et al., 2018). Different types of polycyclic diterpenoid phytoalexins have been identified from the leaves of rice that were either infected with the rice leaf blast pathogen, including oryzalexin S, momilactones A and B (Nemoto et al., 2004). In *S. viridis* leaves, eight enzyme-related genes that catalyze phytoalexin synthesis were significantly downregulated (Figure 7). In summary, reduction of the pathogen resistance of *S. viridis* leaves is also an important pathway of CA stress.

Plant hormones mediate stress responses and developmental pathways together with other signaling molecules, and MAPK plays an indispensable regulatory role in this process (Zhang et al., 2018). ET and JA are two important plant defense hormones that can cooperate to regulate the stress response of plants (Bian et al., 2020). Under CA stress, MAPK cascade kinase genes (*MKK3*, *MPK3/6*), which act as upstream activators in the signaling pathway, were obviously downregulated (Figure 6B). Meanwhile, *SvEIN3* and *SvMYC2*, as important transcription factors in the ET and JA signaling pathways (Pires and Dolan, 2010; Bian et al., 2020), were also significantly downregulated. In addition, expression of ET response genes that depend on EIN3 activation, including *SvERF1* and *SvChiB*, was significantly decreased. These results suggested that CA jointly affected the gene regulation of ET and JA defense responses and reduced the stress resistance of *S. viridis* leaves. ABA is also an important hormone signaling molecule used by plants to resist external threats. The key regulatory mechanism depends on ABA binding with its receptor, PYR/PYL, to inhibit the activity of the negative regulatory factor PP2C, which can reduce the inhibition of *SnRK2* kinase by *PP2C* (Takahashi et al., 2020). In this study, the downregulation of *PYR/PYL* and *PP2C* genes expression inhibited the adaptation of ABA to CA pressure (Figure 7). Direct proof of MAPK activation by ABA was obtained by Ortiz-Masia et al. (2007). It is revealed that MPK1 and MPK2 may mediate ABA signaling in plant cells. However, their upstream activators *SvMAPKKK17/18* and *SvMKK3* have been shown to be underexpressed. In addition, ABA has been reported to induce ROS production during stomatal closure (Zhang et al., 2018). Downregulation of *MPK6* activated in downstream impaired the expression of the catalase gene CAT1 in leaves. This result was consistent with the decrease in CAT activity. In conclusion, CA could downregulate the signal transduction pathway of hormones and weaken the stress resistance of plants, thus inhibiting their growth and development.

## Conclusion

*Artemisia argyi* is an ecologically dominant population in the wild, with strong ecological competitiveness, indicating that it may exist a strong allelopathic effect. Indeed, the aqueous extract of *A. argyi* showed an inhibitory effect on the growth of four common harmful weeds, including monocotyledons and dicotyledons. The identification and quantification of 5-CQA, 3-CQA, 4-CQA, and CA, as well as the allelopathic activity assay of these compounds, suggested that CA might be an important ecological weapon for *A. argyi* to inhibit the growth of other plants and that 5-CQA, 3-CQA, and 4-CQA could assist in its action. Moreover, CA demonstrated significant suppression of *S. viridis* leaf growth in the present study and could cause a functional imbalance in the antioxidant system by inducing ROS production, leading to lipid peroxidation, and disrupting antioxidative enzyme activities. Importantly, CA also exerted allelopathic activity by inhibiting the expression of plant hormone-related genes, especially genes involved in GA and phytoalexin biosynthesis and the ET, JA, and ABA signal transduction. In summary, this study reveals for the first time that *A. argyi* has an ecological advantage because it can secrete a large number of allelochemicals to inhibit the hormone synthesis and function of other plants. In addition, *A. argyi* and caffeic acid could be potential herbicides to control and mitigate harmful weeds.

## Data Availability Statement

The original contributions presented in the study are publicly available. This data can be found here: https://www.ncbi.nlm.nih.gov/bioproject/PRJNA775669/.

## Author Contributions

LC, HD, and DL conceived and designed the project. DL provided financial support. LC and JL collected the samples and performed the experiments. YZ, LuG, and RJ analyzed the data. YM and LaG contributed reagents, materials, and analysis tools. LC wrote the manuscript. All authors approved the final version.

## Conflict of Interest

The authors declare that the research was conducted in the absence of any commercial or financial relationships that could be construed as a potential conflict of interest.

## Publisher’s Note

All claims expressed in this article are solely those of the authors and do not necessarily represent those of their affiliated organizations, or those of the publisher, the editors and the reviewers. Any product that may be evaluated in this article, or claim that may be made by its manufacturer, is not guaranteed or endorsed by the publisher.

## Footnotes

1
http://www.geneontology.org/

2
https://www.kegg.jp/
