# Supplementary material for: Caffeic Acid, an Allelochemical in Artemisia argyi, Inhibits Weed Growth via Suppression of Mitogen-Activated Protein Kinase Signaling Pathway and the Biosynthesis of Gibberellin and Phytoalexin
Source: Front Plant Sci. 2022 Jan 6;12:802198. doi: 10.3389/fpls.2021.802198 (PMC8770944; doi:10.3389/fpls.2021.802198)
Supplement: Supplementary file 1 [file Data_Sheet_1.docx]

Table S1 Primer sequences of reference and target genes used for RT-qPCR.

| Gene | Description | Locus/Gene ID* | Primer | Sequence (5’-3’) |
| --- | --- | --- | --- | --- |
| *IF4A* | Initiation factor 4A | LOC101761364 | *IF4A*_qF | ATTGGTCGTAGTGGTCGTTTC |
|  |  |  | *IF4A*_qR | CCACGTTGTAGAACCTCTGTATG |
| *LOC117837121* | ent-copalyl diphosphate synthase | LOC117837121 | *LOC117837121*_qF | AGGCAAAGAGAAGCCGAAGG |
|  |  |  | *LOC117837121*_qR | GAGGCATCCTGTAGAGCGTC |
| *LOC117850476* | ent-kaurene synthase | LOC117850476 | *LOC117850476*_qF | GCATGGCATGGAGCAAGATG |
|  |  |  | *LOC117850476*_qR | TAGATGGCGCGGAAAACGAT |
| *GA3* | ent-kaurene oxidase | LOC117851887 | *GA3*_qF | CAACAGGCTTTACCCGTTGC |
|  |  |  | *GA3*_qR | TTTCCACGGACGAAAACCCT |
| *GA2ox* | gibberellin 2beta-dioxygenase | LOC117855565 | *GA2ox*_qF | ATTCGATCACAGCTGCCCAA |
|  |  |  | *GA2ox*_qR | CCATAAGCAACAGAGCATGGC |
| *KSL8* | stemar-13-ene synthase | LOC117842731 | *KSL8*_qF | GGCTGTCTACGACACCAACA |
|  |  |  | *KSL8*_qR | CTCACCAAGGCAAGCCAAAC |
| *CYP99A2/3* | 9beta-pimara-7,15-diene oxidase | LOC117842511 | *CYP99A2/3*_qF | AGCATGTTTGGTGACCCTCA |
|  |  |  | *CYP99A2/3*_qR | CTAATCCATCGGAGACCGCT |
| *MAS* | momilactone-A synthase | LOC117861293 | *MAS*_qF | TCTTGTGCACGTGAGAACCG |
|  |  |  | *MAS*_qR | TCTACTCGGTGGAATCGTGGA |
| *MPK3* | mitogen-activated protein kinase 3 | LOC117837485 | *MPK3*_qF | GACGCCTTCGACCTATCATCA |
|  |  |  | *MPK3*_qR | GTTGAAGGTGAGCATCCGCT |
| *EIN3* | ethylene-insensitive protein 3 | LOC117865617 | *EIN3*_qF | GCGGAGAACACACCGACTAT |
|  |  |  | *EIN3*_qR | CCGCCTCAGAGAAAGGATGC |
| *ERF1* | ethylene-responsive transcription  factor 1 | LOC117850528 | *ERF1*_qF | GGGAGGAAAGATCAAGGGGC |
|  |  |  | *ERF1*_qR | TGGTGCGAACAAGTAGTCCC |
| *ChiB* | basic endochitinase B | LOC117833644 | *ChiB*_qF | CGGCGATCGTGACTGGTAAT |
|  |  |  | *ChiB*_qR | CGACAGCCTTCCTTCACACT |
| *MKK3* | mitogen-activated protein kinase  kinase 3 | LOC117836726 | *MKK3*_qF | TTCGGGTGCTTCAGACCATC |
|  |  |  | *MKK3*_qR | CATAAGCCCGTGCCATCCAA |
| *MPK6* | mitogen-activated protein kinase 6 | LOC117837957 | *MPK6*_qF | GCCTTTAACCCGGATCCTCC |
|  |  |  | *MPK6*_qR | CAACAACCCATGTCCGTGTG |
| *MYC2* | transcription factor MYC2 | LOC117850680 | *MYC2*_qF | GGAGATAGGGGACGTCAGGA |
|  |  |  | *MYC2*_qR | AACGTTAAGGTGGTGGCTCA |
| *PYL* | abscisic acid receptor PYR/PYL family | LOC117839701 | *PYL*_qF | TGCTGGTACTGGATGAGGGA |
|  |  |  | *PYL*_qR | AGGAACACACGATCAACACGA |
| *PP2C* | protein phosphatase 2C | LOC117848121 | *PP2C*_qF | TGAGCATGTAGCTGTAGCGG |
|  |  |  | *PP2C*_qR | GACACCGAGAGCCTCAAACA |
| *MAPKKK17_18* | mitogen-activated protein kinase kinase kinase 17/18 | LOC117856468 | *MAPKKK17_18*_qF | TTGACGTGGAAGACGCCATT |
|  |  |  | *MAPKKK17_18*_qR | CAAGGACAACCGAGTTCCGA |

**Setaria viridis* locus/gene identifiers; F, forward; R, reverse.

Table S2 Identification of chemical components in the *A. argyi* aqueous extract.

| No. | RT（min） | m/z [M-H]^-^ | Molecular formula | MS/MS | Proposed compound |
| --- | --- | --- | --- | --- | --- |
| 1 | 0.56 | 191.0562 | C_7_H_12_O_6_ | 173.0423, 93.0332, 87.0075, 85.0286, 59.0115 | Quinic acid |
| 2 | 2.65 | 153.0538 | C_8_H_10_O_3_ | 135.0434 | Hydroxytyrosol |
| 3 | 2.91 | 353.0862 | C_7_H_12_O_6_ | 191.0562, 179.0358, 173.0470, 161.0217, 135.0434 | Neochlorogenic acid (5-CQA) |
| 4 | 4.02 | 353.0862 | C_7_H_12_O_6_ | 191.0562, 179.0358, 173.0423, 161.0262, 135.0434 | Chlorogenic acid (3-CQA) |
| 5 | 4.24 | 353.0862 | C_7_H_12_O_6_ | 191.0562, 179.0358, 173.0423, 161.0217, 135.0434 | Cryptochlorogenic acid (4-CQA) |
| 6 | 4.40 | 179.0358 | C_9_H_8_O_4_ | 135.0434, 134.0346 | Caffeic acid (CA) |
| 7 | 4.51 | 305.0671 | C_12_H_18_O_7_S | 225.1132, 96.9589, 59.0115 | Hydroxyjasmonic acid-O-sulphate |
| 8 | 6.12 | 563.1377 | C_26_H_28_O_14_ | 503.1229, 473.1072, 443.0951, 383.0797, 353.0663 | Schaftoside |
| 9 | 7.51 | 515.1190 | C_25_H_24_O_12_ | 353.0862, 335.0746, 191.0562, 179.0358, 173.0423, 161.0217, 135.0434 | Isomer of isochlorogenic acid B |
| 10 | 7.83 | 515.1190 | C_25_H_24_O_12_ | 353.0862, 335.0746, 191.0562, 179.0358, 173.0423, 161.0217, 135.0434 | Isochlorogenic acid A |
| 11 | 7.91 | 515.1111 | C_25_H_24_O_12_ | 353.0862, 335.0746, 191.0562, 179.0358, 173.0423, 161.0217, 135.0434 | Isomer of isochlorogenic acid A |
| 12 | 8.47 | 515.1190 | C_25_H_24_O_12_ | 353.0862, 335.0746, 191.0562, 179.0311, 173.0423, 135.0434 | Isochlorogenic acid C |
| 13 | 12.64 | 329.0627 | C_17_H_14_O_7_ | 314.0433, 299.0153 | Jaceosidin |
| 14 | 14.69 | 343.0817 | C_18_H_16_O_7_ | 328.0544, 313.0333, 298.0115, 132.0244 | Eupatilin |

Note: RT denotes retention time.

Table S3 Quantitative analysis of chemical constituents in the *A. argyi* aqueous extract.

| No. | Name | Standard curve | Concentration |
| --- | --- | --- | --- |
| 1 | 5-CQA | y=4198.904x+0.049 R^2^=0.9998 | 0.41 ±0.01 mg·g^-1^ |
| 2 | 3-CQA | y=3975.955x-0.099 R^2^=0.9993 | 0.19 ±0.01 mg·g^-1^ |
| 3 | 4-CQA | y=2807.421x+0.072 R^2^=0.9994 | 0.42 ±0.01 mg·g^-1^ |
| 4 | CA | y=6871.704x+0.207 R^2^=0.9998 | 1.52 ±0.01 mg·g^-1^ |
| 5 | TPC | y = 0.0089x + 0.0648 R^2^ = 0.9991 | 78.40 ± 1.28 mg GAE/g Fr. |
| 6 | TFC | y = 0.0061x + 0.0405 R^2^ = 0.9996 | 30.19 ± 0.19 mg RuE/g Fr. |

Note: The TPC and TFC represent the contents of total phenolic acid and total flavonoid, respectively.

Table S4 Read Summary statistics of mRNA sequencing

| No. | Sample name | Total Raw Reads | Total Clean Reads | Total Clean Bases (Gb) | Total Mapping (%) | Total data ≥ Q30 (%) | GC content (%) |
| --- | --- | --- | --- | --- | --- | --- | --- |
| 1 | CK-1 | 43821050  43821050  43821050  43821050  43821050  43821050 | 42408416 | 6.36 | 92.62 | 92.91 | 53.38 |
| 2 | CK-2 | 43821050 | 42551180 | 6.38 | 92.19 | 92.15 | 53.55 |
| 3 | CK-3 | 43821050 | 42723002 | 6.41 | 92.80 | 92.53 | 53.50 |
| 4 | CA-1 | 43821050 | 42459744 | 6.37 | 93.11 | 92.48 | 53.28 |
| 5 | CA-2 | 43821050 | 42779598 | 6.42 | 93.34 | 92.56 | 53.15 |
| 6 | CA-3 | 43821050 | 42024334 | 6.30 | 92.59 | 92.74 | 53.60 |
